# Impact of lifestyle and clinical factors on the prognosis of tennis elbow

**DOI:** 10.1038/s41598-024-53669-x

**Published:** 2024-02-06

**Authors:** Chan Zhang, Zhongwei Jia, Jiangbo Li, Xu Wang, Shengping Yang

**Affiliations:** 1 College of Physical Education, Shanxi University of Finance and Economics, Sports Industry Research Center of Shanxi University of Finance and Economics, Taiyuan, Shanxi China; 2 Department of Orthopedic, Shanxi Provincial People’s Hospital, Taiyuan, Shanxi China; 3 Department of Orthopedic, Taiyuan Central Hospital, JieFang Street, Dongsandao 1#, Taiyuan, 030001 Shanxi China; 4 Department of Literature Search, Shanxi Research Center for Information and Strategy of Science and Technology, Taiyuan, Shanxi China

**Keywords:** Health care, Medical research, Risk factors

## Abstract

Tennis elbow (lateral epicondylitis) typically responds well to conservative treatment, and few patients require surgical intervention. This study aimed to investigate the influence of lifestyle and clinical factors on the prognosis of tennis elbow. This prospective, multicenter, nested case–control study included patients diagnosed with lateral epicondylitis after excluding other conditions. Patients who required surgery because of inadequate improvement after 6 months of conservative treatment were defined as the case group; the remaining patients constituted the control group. Propensity score matching was performed to eliminate baseline differences. Univariate and multivariate analyses were performed using logistic regression. This study included 265 patients (53 in the case group, 212 in the control group). Multivariate analysis revealed that smoking, alcohol consumption, and frequent physical exercise were independent risk factors for surgical intervention, whereas combined treatment with oral nonsteroidal anti-inflammatory drugs (NSAIDs) and local corticosteroid injections was a protective factor against surgery. Subgroup analysis showed that heavy drinkers had a 3.74-fold higher risk of requiring surgical treatment within 1 year than occasional drinkers. Smoking and alcohol consumption were associated with non-operative treatment failure in patients with lateral epicondylitis. Combining oral NSAIDs and corticosteroid injections is a favorable conservative treatment option.

## Introduction

Tennis elbow, also known as lateral epicondylitis, is a common condition affecting the elbow joint. Although this condition was originally associated with playing tennis due to its prevalence in tennis players, current evidence shows that tennis-associated injuries account for only 5% of cases^1^. The hallmark symptom of this condition is self-limiting pain on the outer aspect of the elbow that becomes aggravated during wrist extension and forearm rotation.

Two main theories exist regarding the pathogenesis of tennis elbow: tendon degeneration theory and inflammatory mediator theory^2^. The tendon degeneration theory posits that tendon damage results from overuse, leading to collagen degeneration, microcirculation disturbance, and fibroblast proliferation independent of the presence of inflammatory mediators^3,4^. In contrast, the inflammatory mediator theory suggests that overuse of the tendon leads to the release of inflammatory mediators, driving tendon degeneration and playing a pivotal role in the pathological process of the disease^5^.

The clinical management of tennis elbow includes conservative and surgical approaches^6^. Conservative treatments include physical therapy, rehabilitation, oral administration of nonsteroidal anti-inflammatory drugs (NSAIDs), and local corticosteroid injections. Approximately 90% of patients experience considerable symptom improvement within 1 year of conservative treatment. Surgical intervention is required in only 5–10% of patients who poorly respond to conservative treatment and develop chronic symptoms^7^. The most common treatment approaches include oral NSAID use and local corticosteroid injections. However, there is no consensus on whether NSAIDs or corticosteroids can reduce the risk of failure of nonoperative treatments associated with tennis elbow^8,9^.

Lifestyle factors such as smoking, alcohol drinking, and dietary habits are known to influence the prognosis of various medical conditions. Smokers showed a higher probability of developing lateral epicondylitis compared to non-smokers^10,11^. Otoshi et al. and Hyung et al. suggested that alcohol intake was not significantly associated with lateral epicondylitis^12,13^. However, to date, no studies have explored the effects of smoking and alcohol consumption on the prognosis of lateral epicondylitis. Considering that China is one of the countries with the largest consumptions of alcohol and tobacco in the world, the effects of smoking and alcohol consumption on the prognosis of tennis elbow must be investigated. Therefore, we conducted a prospective, nested case–control study to examine the impact of lifestyle and clinical factors on the prognosis of tennis elbow.

## Results

After the inclusion and exclusion criteria were applied, 620 patients were enrolled in this study, comprising 53 patients who underwent surgical treatment and 567 patients who received conservative treatment (surgical treatment rate, 8.55%). Statistical analysis showed significant differences in VAS pain scores and age between the two groups. PSM was used to mitigate these statistical differences. After matching, 53 and 212 patients constituted the case and control groups, respectively. The patients’ baseline information is presented in Table 1. The proportion of smokers was 50.94% in the control group and 77.36% in the case group. Furthermore, 50.47% of patients in the control group and only 28.30% in the case group received oral NSAIDs as treatment. In the case group, a higher proportion of patients (30.19%) received combined treatment with oral NSAIDs and local corticosteroid injections.

**Table 1:** Baseline information.

Variable	Control group (*N* = 212)	Case group (*N* = 53)	*P*
Sex, *n* (%)			
Female	74 (34.91)	15 (28.30)	0.363
Male	138 (65.09)	38 (71.70)	
Age (years)	49 [43, 56]	47 [41, 58]	0.464
BMI (kg/m^2^)	22.89 [20.83, 24.84]	23.15 [20.48, 25.91]	0.894
VAS score	2 [2, 3]	2 [2, 3]	0.183
Smoking, *n* (%)			
Yes	108 (50.94)	41 (77.36)	< 0.001*
No	104 (49.06)	12 (22.64)	
Alcohol consumption, *n* (%)			
Yes	139 (65.57)	43 (81.13)	< 0.001*
No	73 (34.43)	10 (18.87)	
Coffee consumption, *n* (%)			
Yes	39 (18.40)	10 (18.87)	0.937
No	173 (81.60)	43 (81.13)	
Insomnia, *n* (%)			
Yes	70 (33.02)	19 (35.85)	0.696
No	142 (66.98)	34 (64.15)	
Physical exercise, *n* (%)			
Never	80 (37.74)	22 (41.51)	0.006*
Occasional	114 (53.77)	19 (35.85)	
Frequent	18 (8.49)	12 (22.64)	
Diet, *n* (%)			
High-fat diet	46 (21.70)	14 (26.42)	0.002*
High-carbohydrate diet	157 (74.06)	30 (56.60)	
Balanced diet	9 (4.25)	9 (16.98)	
Underlying disease, *n* (%)			
None	168 (79.25)	43 (81.13)	0.524
Hypertension	26 (12.26)	7 (13.208)	
Diabetes	1 (0.47)	1 (1.89)	
Hyperlipidemia	17 (8.02)	2 (3.77)	
Treatment, *n* (%)			
Oral NSAIDs	107 (50.47)	15 (28.30)	< 0.001*
Local corticosteroid injections	27 (12.74)	3 (5.66)	
Physical therapy	16 (7.55)	11 (20.76)	
Oral NSAIDs + local corticosteroid injections	33 (15.57)	16 (30.19)	
NSAIDs + physical therapy	3 (1.42)	3 (5.66)	
NSAIDs + corticosteroid injections + physical therapy	11 (5.19)	4 (7.55)	
Other conservative treatment methods	15 (7.08)	1 (1.89)	

*P < 0.05; *BMI* body mass index.

Univariate analysis demonstrated statistically significant differences in smoking status, alcohol consumption, physical exercise, diet, and treatment type (Table 2). These variables were subsequently included in the final model. Multivariate analysis demonstrated (Table 3) that smoking (OR 4.20, 95% CI 1.91–9.98, *P* = 0.002), alcohol consumption (OR 17.32, 95% CI 7.15–48.50, *P* < 0.001), and frequent physical exercise (OR 1.94, 95% CI 1.69–5.40, *P* = 0.036) were independent risk factors for non-surgical treatment failure, whereas combined treatment with oral NSAIDs and local corticosteroid injections was a protective factor against surgery (OR 0.45, 95% CI 0.31–0.94, *P* = 0.046).

**Table 2:** Univariate analysis by conditional logistic regression.

Variable	OR	95% CI	*P*
BMI	1.01	0.93–1.09	0.818
Smoking	3.29	1.64–6.61	0.001*
Alcohol consumption	8.19	3.89–17.23	< 0.001*
Coffee consumption	1.03	0.48–2.23	0.937
Insomnia	1.13	0.60–2.13	0.696
Physical exercise			
Occasional	0.61	0.31–1.19	0.147
Frequent	2.42	1.02–5.78	0.046*
Diet			
High-fat diet	0.63	0.31–1.28	0.202
High-carbohydrate diet	3.29	1.09–9.88	0.034*
Underlying disease			
Hypertension	1.05	0.43–2.59	0.912
Diabetes	3.91	0.23–63.74	0.339
Hyperlipidemia	0.46	0.10–2.07	0.311
Treatment			
Local corticosteroid injections	0.79	0.21–2.94	0.728
Physical therapy	4.90	1.92–12.54	0.001*
Oral NSAIDs + local corticosteroid injections	0.46	0.35–0.74	0.003*
NSAIDs + physical therapy	7.13	1.32–38.62	0.023*
NSAIDs + corticosteroid injections + physical therapy	2.59	0.73–9.20	0.140
Other conservative treatment methods	0.48	0.06–3.87	0.487

*P < 0.05.

**Table 3:** Results of multivariate analysis by conditional logistic regression.

Variable	OR	95% CI	*P*
Smoking	4.20	1.91–9.98	0.002*
Alcohol consumption	17.32	7.15–48.50	< 0.001*
Physical exercise			
Occasional	0.60	0.28–1.27	0.183
Frequent	1.94	1.69–5.40	0.036*
Diet			
High-fat diet	0.67	0.29–1.60	0.356
High-carbohydrate diet	2.48	0.65–9.87	0.185
Treatment			
Local corticosteroid injections	0.82	0.17–3.03	0.783
Physical therapy	1.93	0.56–6.31	0.282
Oral NSAIDs + local corticosteroid injections	0.45	0.31–0.94	0.046*
NSAIDs + physical therapy	4.44	0.69–28.45	0.103
NSAIDs + corticosteroid injections + physical therapy	3.58	0.82–13.91	0.072
Other conservative treatment methods	0.63	0.03–4.09	0.678

*P < 0.05.

Our results suggest that alcohol consumption is associated with a significantly increased risk of non-surgical treatment failure. Consequently, we conducted a subgroup analysis involving patients with alcohol consumption to investigate the impact of different alcohol consumption levels on patient outcomes. The subgroup analysis revealed that heavy drinkers had a 3.74-fold higher risk of requiring surgery within 1 year than occasional drinkers (OR 3.74, 95% CI 2.27–5.12, *P* = 0.016) (Table 4).

**Table 4:** Subgroup analysis in patients with alcohol consumption.

Variable	OR	95% CI	*P*
Moderate drinking	1.31	0.97–1.93	0.088
Heavy drinking	3.74	2.27–5.12	0.016*

*P < 0.05.

## Discussion

To our knowledge, this is the first report on prognostic risk factors for lateral epicondylitis in a Chinese population. Our study is the first to identify a correlation between smoking and heavy alcohol consumption and an unfavorable prognosis for tennis elbow. Considering that lateral epicondylitis typically occurs during the working years and that it could affect a patient’s ability to work and live normally, this study holds significant social value and clinical importance.

According to data from the Chinese government, as many as 350 million individuals in China are smokers. Smoking is known to be associated with various diseases, such as coronary heart disease and lung cancer. Tobacco smoke contains various chemicals, including numerous reactive oxygen and nitrogen species that can induce inflammatory responses and even cell apoptosis^14^. The pathogenesis of lateral epicondylitis may be related to inflammatory responses^5^. Our study demonstrates that smoking substantially negatively influences patients’ recovery from lateral epicondylitis. Smokers in this study had a 4.2-fold higher risk of undergoing surgery than nonsmokers, this indicates the dangers of smoking.

In China, alcohol is not only a beverage but also a social commodity. Most adult men engage in some extent of alcohol indulgence, drinking mainly spirits, followed by beer and, less frequently, wine. Long-term alcohol consumption can lead to alcohol-related disorders such as liver disease and brain damage. Our research reveals that alcohol consumption is an independent risk factor for poor treatment outcomes in patients with lateral epicondylitis. Particularly, heavy drinkers are 3.74 times more likely to require surgery within 1 year than occasional drinkers. We speculate that alcohol-induced inflammatory reactions contribute to disease progression.

In muscle, bone, and physical and sports medicine, exercise is considered the most effective treatment for chronic symptoms of lateral epicondylitis. However, no clear evidence exists on which specific exercise regimen (e.g., exercise type, duration, and frequency) benefits recovery from this condition. Our experimental findings were rather unexpected: compared with individuals who abstain from exercise, those who engage in frequent physical exercise not only failed to experience improved recovery but also had an increased likelihood of requiring surgical intervention. The prevailing belief is that lateral epicondylitis is often a result of excessive muscle use, which triggers the proliferation of tendon cells and matrix material^7^. Consequently, conservative treatment often involves minimizing use of the affected area. We hypothesize that certain exercise regimens adopted by a subset of our study patients may have inadvertently exacerbated damage to the affected area, leading to the development of a stubborn case of tennis elbow. However, as our study did not gather data on the type, duration, or frequency of physical exercise performed by the patients, caution should be taken when interpreting our results on the relationship between physical exercise and the prognosis of lateral epicondylitis.

Another contentious topic within this domain pertains to the potential long-term benefits of corticosteroid injections in the presence of degenerative changes. Our results suggest that, compared with oral NSAIDs alone, combined treatment with oral NSAIDs and local corticosteroid injections can reduce the likelihood of requiring surgical treatment. Inflammatory changes are often absent in the early stage of lateral epicondylitis^15^. We speculate that patients with middle to late-stage tennis elbow exhibit inflammatory changes, and inflammatory diseases respond well to corticosteroid treatment. In our hospital, most patients achieve favorable outcomes with NSAIDs alone, and corticosteroid injections are reserved for those who do not adequately respond to NSAID monotherapy. This may explain the reason why the combination of oral NSAIDs and corticosteroid injections can improve the prognosis of patients with lateral epicondylitis.

The primary strength of this study is its prospective design, which minimizes recall bias. Nonetheless, several limitations should be acknowledged. First, the relatively small number of patients in the case group may affect the interpretation of results. Second, it is important to recognize that various stages of the condition may necessitate different treatment approaches, although we did not make these finer distinctions. Third, patients may be incorrectly grouped solely based on the surgery status. Lastly, the potential influence of different exercise regimens on disease progression was not accounted for because we did not collect this information from patients.

In conclusion, for patients with lateral epicondylitis, smoking and alcohol consumption were associated with non-operative treatment failure. Combining oral NSAIDs and local corticosteroid injections is a viable conservative treatment option.

## Methods

### Cohort description

The current analysis is a prospective non-interventional clinical study. This nested case–control study included orthopedic patients at Taiyuan Central Hospital and Shanxi Provincial People’s Hospital, both tertiary teaching hospitals with > 2000 beds. Between March 2020 and September 2022, 620 adult patients (aged > 18 years) who met the inclusion / exclusion criteria and signed the informed consent were enrolled in this study. This study was approved by the ethics committee of Shanxi Provincial People’s Hospital, Shanxi Province, Taiyuan, China (approval no. 202036).

### Definitions

The diagnostic criteria for lateral epicondylitis were as follows^15,16^:

1. Insidious onset with apparent injury or provoking factors.
2. Pain on the lateral aspect of the elbow, precisely at the lateral epicondyle, with a localized and tender point; restricted rotation of the wrist and forearm.
3. Absence of cutaneous signs of inflammation.
4. Positive result in the forearm extensor muscle stretch test (Mill’s sign).
5. Exclusion of other conditions such as radial tunnel syndrome, posterolateral elbow instability, anconeus or triceps tendinitis, cervical radiculopathy, synovitis or lateral ligament injury.
6. Only complex cases will be differentiated by radiographs, ultrasonography, or MRI.

Occasional drinking was defined as consuming < 100 ml of strong spirits or < 500 ml of beer weekly. Moderate drinking was defined as consuming 100–500 ml of strong spirits or 500–2500 ml of beer weekly. Excessive drinking was defined as consuming > 500 ml of strong spirits or > 2500 ml of beer weekly. A balanced diet was defined as daily consumption of at least 100 g of vegetables or fruits, with daily fat and oil intake of < 50 g. A high-fat diet was defined as a diet with fat and oil intake of > 50 g daily. A high-carbohydrate diet was defined as a diet primarily consisting of starchy foods (e.g., rice and wheat), with daily vegetable or fruit intake of < 100 g and daily fat and oil intake of < 50 g.

### Data collection

The following information was collected from patients upon their enrollment: sex, age, height, weight, visual analog scale (VAS) score, smoking status (yes or no), alcohol consumption (never, occasional, moderate, or heavy drinking), coffee consumption (yes or no), hypertension (yes or no), diabetes (yes or no), and hyperlipidemia (yes or no). The patients were subsequently followed up by phone every 6 months. During the follow-up assessments, the following information was recorded: smoking status, alcohol consumption, coffee consumption, laboratory tests, treatment received (conservative or surgical treatment), details of conservative treatment (oral NSAIDs, local corticosteroid injections, physical therapy, or other methods), insomnia (yes or no), physical exercise (never, occasional [one to four times per month], or frequent [more than four times per month]), diet (high-fat, high-carbohydrate, or balanced diet), and timing of surgical treatment. The follow-up duration ranged from 12 to 18 months.

### Criteria for selection of cases and controls

The inclusion criteria were age ≥ 18 years and an initial diagnosis of lateral epicondylitis. The exclusion criteria were as follows: inability to complete the follow-up, refusal to provide informed consent, missing data, and surgical treatment within 6 months of diagnosis. All the patients were asked to perform activity modification, avoid painful activities, and undergo rehabilitation once they were diagnosed with lateral epicondylitis. Patients who underwent surgery after the failure of conservative treatment for more than 6 months were defined as the case group, and the remaining patients constituted the control group.

### Statistical analysis

This study was designed as a prospective, nested case–control study. Descriptive statistics were collected from the study cohort, including sex distribution, age, body weight, and height. Shapiro–Wilk test was used to test data normality. Medians and interquartile ranges or means and standard deviations were used as statistical descriptions of quantitative variables. Propensity score matching (PSM) was used to adjust demographic data (VAS score and age). Each case was matched to up to four controls. Risk factors for requiring surgical treatment were analyzed using univariate and multivariate conditional logistic regression models in which the dependent variable was surgical treatment (yes or no) and the main exposure variables were smoking status (yes or no), alcohol consumption (never, occasional, moderate, or heavy drinking), coffee consumption (yes or no), insomnia (yes or no), physical exercise (never, occasional, or frequent), diet (high-fat, high-carbohydrate, or balanced diet), hypertension (yes or no), diabetes (yes or no), and hyperlipidemia (yes or no). The models were used to calculate the OR and 95% confidence interval (CI) of the association between the variables and the risk of requiring surgical treatment.

### Ethics approval and consent to participate

The study protocol was approved by the Ethics Committee of Shanxi Provincial People’s Hospital (202036) and was conducted following the legal requirements and tenets of the Declaration of Helsinki and its subsequent amendments. All enrolled patients signed informed consent.

## Data Availability

The data that support the findings of this study are available from the corresponding author (Professor Jiangbo Li) upon reasonable request.
